# Effect of Aerobic Exercise Training on MDA and TNF-***α*** Levels in Patients with Type 2 Diabetes Mellitus

**DOI:** 10.1155/2014/820387

**Published:** 2014-10-21

**Authors:** Mihriban Arslan, Suleyman Hilmi Ipekci, Levent Kebapcilar, Nesrin Dogan Dede, Sevil Kurban, Ekrem Erbay, Mustafa Sait Gonen

**Affiliations:** ^1^Department of Internal Medicine, Develi State Hospital, 38400 Kayseri, Turkey; ^2^Division of Endocrinology and Metabolism, Faculty of Medicine, Selcuk University, 42250 Konya, Turkey; ^3^Department of Internal Medicine, Faculty of Medicine, Baskent University, 42080 Konya, Turkey; ^4^Department of Biochemistry, Meram School of Medicine, Necmettin Erbakan University, 42090 Konya, Turkey; ^5^Division of Endocrinology and Metabolism, Faculty of Medicine, Istanbul Bilim University, 34394 Istanbul, Turkey

## Abstract

*Objective.* Diabetes mellitus (DM) is associated with low-grade inflammation. The benefits of regular exercise for the DM are well established, whereas less is known about the impact of aerobic exercise on malondialdehyde (MDA) and tumor necrosis factor-alpha (TNF-*α*) in the DM. *Methods.* We randomised 64 participants, who do not exercise regularly, without any diabetic chronic complications in parallel to 12 weeks of aerobic exercise (three times per week, *n* = 31) and no exercise (control; *n* = 33). Plasma levels of soluble TNF-*α* and MDA levels were measured before-after physical training programme and control group. *Results.* Sixty-four patients with type 2 diabetes mellitus were analysed. When comparing the two groups of patients with age, gender, hemoglobin A1c (HbA1c) levels, lipid profile, waist circumference, body mass index (BMI) and class of treatment for diabetes were not different between groups. While soluble TNF-*α* remained essentially unaffected by physical training, plasma concentrations of MDA markedly decreased (*P* < 0.05); physical training also decreased body weight, waist circumference, and blood pressure (*P* < 0.05). *Conclusion.* Exercise training favorably affected body weight, waist circumference, and blood pressure. A three-weekly, 12-week, aerobic-training programme, without a concomitant weight loss diet, was associated with significant decrease in MDA levels in type 2 diabetic individuals.

## 1. Introduction

Malondialdehyde (MDA) and tumor necrosis factor-alpha (TNF-*α*) are important markers which may reflect low-grade systemic inflammation [1]. MDA and TNF-*α* are produced in various tissues under diabetic conditions and these parameters have often been implicated in the pathogenesis of micro and macrovascular diseases observed in diabetic individuals [2, 3].

An association between physical inactivity and low-grade systemic inflammation has been demonstrated in cross-sectional studies [4, 5]. Regular aerobic exercise decreases cardiovascular risk of people with type 2 diabetes mellitus (T2DM) principally by reducing body weight and abdominal visceral fat accumulation with subsequent improvements in insulin sensitivity, blood pressure, lipid profile, and glycemic control [6, 7]. Despite the increasing amount of evidence that shows the benefits of regular aerobic exercise for patients with T2DM, there is only limited information about the effects of aerobic exercise on the expression of MDA and TNF-*α* levels. The effect of exercise on circulating TNF-*α* remains inconclusive; previous studies have shown that its circulating levels are either unchanged or exhibit increments/decrements following exercise [8–11].

We, therefore, sought to investigate the effects of a three-weekly aerobic-training programme, without a concomitant weight loss diet, on circulating levels of plasma biomarkers of MDA and TNF-*α* in type 2 diabetic patients, who were free of known cardiovascular disease.

## 2. Methods

### 2.1. Participants

The study was approved by the ethical committees of the Meram Faculty of Medicine at Selcuk University. T2DM was defined according to the American Diabetes Association criterion [12].

Seventy-five T2DM patients aged between 35 and 70, with HbA1c of 6.5% or above, were assessed for eligibility. Patients with stable T2DM, all free of exercise limiting comorbidities, such as cerebrovascular disease, musculoskeletal impairment, or vascular disease of the lower extremities, were enrolled in this study. Potential participants were excluded if they (1) were receiving insulin therapy, (2) were participating in exercise ≥2 times per week for at least 20 min per session or had been in any resistance training during the previous 6 months, (3) reported changes in oral hypoglycemic medications during the previous 2 months, (4) reported changes in antihypertensive or lipid lowering agents in the previous month, (5) reported a change of ≥5% in body weight during the previous 2 months, (6) had serum creatinine ≥200 *μ*mol/L, (7) had proteinuria >1 g/24 h, (8) had blood pressure >160/95 mmHg, (9) had restrictions in physical activity due to disease, (10) had other medical conditions making participation inadvisable, (11) completed fewer than ten of the 12 scheduled exercise sessions during the study, (12) had HbA1c > 7.5%, (13) were smoking, and (14) have known microvascular and/or macrovascular diseases. All participants gave informed written consent.

Subjects were planned into two groups: aerobic exercise training and no exercise. They were medical outpatients. Of these, 9 were unwilling to participate following completion of the study preparation period (4 patients refused exercise place, 2 patients refused dietary advices, 3 patients refused to be a control group). Participants were randomly allocated in equal numbers to the aerobic and no exercise (control) groups. During the study period, 2 patients dropped out (2 in aerobic exercise) due to noncompliance and lost follow-up. Sixty-four patients were treated with oral medication. The medications for diabetes mellitus, lipid profile, and hypertension were not changed during the study.

The aerobic exercise group, which consisted of 31 subjects, did exercise three days a week. Participants exercised three times per week, with training progressing gradually in length and intensity. The aerobic group exercised on treadmills and/or bicycle ergometers. Heart rate monitors were used to adjust workloads to achieve target heart rate. Participants progressed from 15 to 20 min per session at 60% of maximum heart rate to 45 min per session at 75% of measured maximum heart rate. A personal trainer was present for a minimum of three scheduled sessions weekly at each exercise facility and supervised exercise programme. After randomisation, he/she met each participant individually at least once a week for 4 weeks, every 2 weeks for the subsequent 2 months to ensure appropriate progression through the programme. The personal trainer monitored attendance and contacted the participant if any sessions were missed. Control participants were asked to revert to their prestudy activity levels. The control group continued with their normal daily activities during the twelve weeks of research without additional guided physical activities. Efforts were made to minimise dietary and medication cointervention.

### 2.2. Laboratory Tests

All blood samples were obtained in the morning between 08:00 and 09:00 h after an overnight fast in all subjects. Measurements were taken on recruitment and repeated after twelve weeks. The samples were centrifuged, aliquoted, and immediately frozen at −80°C for analyses of MDA and TNF-*α* level.

Serum glucose, total cholesterol, LDL-cholesterol, triglyceride, and HDL-cholesterol levels were measured using Randox enzymatic kits in Roche-Hitachi Modular system. LDL-cholesterol was calculated by the Friedewald equation method.

Plasma MDA levels were determined by using the methods of Draper and Hadley based on thiobarbituric acid (TBA) reactivity [13]. In this method, firstly, 2.5 mL of 10% trichloroacetic acid and 0.5 mL of plasma were added into tube and mixed. After incubating for 15 min at 90°C and cooling with cold water the mixture was centrifuged at 3000 rpm for 10 min. Thereafter, 2 mL of the supernatant was added to 1 mL of 0.675% TBA solution in a test tube. The tube was sealed and incubated at 90°C for 15 min and then cooled to room temperature. The optical density was measured at 532 nm by a spectrophotometer. MDA was expressed as nmol/mL. TNF-*α* plasma concentration was assayed by commercially available ELISA kits (Bender MedSystems Diagnostics, Vienna, Austria) and expressed in pg/mL.

Body weight (kg) and height (cm) were measured. The body mass index (BMI) was calculated as weight in kilograms divided by the square of height in meters. Waist circumference was measured as the minimum between the costal margin and iliac crest. Physical examination including systolic blood pressure and diastolic pressure measurement were done using a mercury sphygmomanometer after 10 minutes of rest.

### 2.3. Statistical Analysis

Results are expressed as mean ± SD. To compare continuous variables distribution, we used Student's *t*-test. Differences between categorical variables were analysed with the *χ* ^2^ test. Pre- and postexercise group values of the parameters were compared with a paired sample *t*-test. The relationships between different variables were analysed with Pearson correlation test. The statistical analysis was carried out by using Statistical Package for the Social Sciences (SPSS), version 16.0 (SPSS, Chicago, IL). A *P* value of <0.05 was considered to be statistically significant.

## 3. Results

A total of 64 patients, comprising 33 females and 31 males, were included in the analysis. Aerobic exercise and control groups did not differ on baseline HbA1c, age, gender, duration of diabetes, BMI, waist circumference, systolic and diastolic arterial pressure, low density lipoprotein cholesterol (LDL-c), high density lipoprotein cholesterol (HDL-c), triglyceride (TG), total cholesterol (TC), MDA, and TNF-*α* levels (Table 1; *P* > 0.05).

HbA1c levels were decreased but missed the significance in aerobic exercise group (6.8 ± 0.8 versus 6.6 ± 1.0, *P* > 0.05). Exercise training group (*n* = 31) completed the follow-up during twelve weeks, with a percentage of weight loss of 2.4%. BMI, waist circumference, and systolic and diastolic arterial pressure were significantly decreased after a 12-week period in exercise training group (Table 2; *P* < 0.05). The levels of MDA statistically significantly decreased whereas TNF-*α* levels did not change significantly after the 12-week period in exercise training group (Table 2; *P* < 0.05).

Clinical and laboratory characteristics and the results of comparisons in control group are given in Table 2. HbA1c, MDA, TNF-*α*, lipid profile, and systolic and diastolic arterial pressure did not differ significantly after the 12-week period (Table 2; *P* > 0.05).

Pearson's correlation analysis revealed that baseline MDA levels were correlated with diastolic arterial pressure in the exercise training group. After 12-week period in aerobic exercise group, Pearson's correlation analysis revealed that MDA levels were only correlated with diastolic arterial pressure (*r* = 0.374; *P* = 0.038) and TNF-*α* levels were positively correlated with LDL-C levels (*r* = 0.374, *P* = 0.046).

## 4. Discussion

We found that the 12-week aerobic exercise training program improved metabolic factors, such as BMI, waist circumference, and systolic and diastolic arterial pressure in sedentary type 2 diabetic group. These results suggest that aerobic exercise training program is beneficial for type 2 diabetic subjects. The impact of aerobic exercise training on oxidative stress in patients with T2DM mellitus has not been fully investigated. Our data show that an aerobic exercise training program did induce significant changes in MDA levels in type 2 diabetic subjects maintaining their usual dietary habits.

Oxidative stress is commonly considered to have occurred if there is a decrease in concentration or activity of nonenzymatic or enzymatic antioxidants or an increase in oxidation of nonenzymatic antioxidants. Oxidative stress has been implicated in the accelerated atherosclerosis and microvascular complications of DM. In previous reports, TBARs and lipid peroxides (as reflect oxidative stress) were found to be elevated in diabetic patients with microvascular complications compared to diabetic patients without microvascular complications [14, 15]. Furthermore, physical exercise may acutely induce oxidative damage, although regular training appears to enhance antioxidant defenses and, in some animal studies, has decreased lipid peroxidation. Aerobic exercise training can reduce oxidative stress by enhancing antioxidant defense mechanisms that include antioxidant enzymes such as superoxide dismutase, catalase, and glutathione peroxidase [16]. Based on the results of our study, we propose that endurance exercise training over twelve weeks is important for reducing parameters of oxidative stress.

Improvement in glycemic control was found to be the critical factor in reducing the risk of chronic diabetic complications. HbA1c levels were decreased but missed the significance in aerobic exercise group. The lack of a statistically significant decrease in A1c levels may be due to the limited number of participants, the duration of training, and the good overall baseline metabolic control in our sample as reflected by a mean baseline A1c <7.5% in aerobic exercise group. HbA1c also provides an integrated measurement of blood glucose during the previous 2 to 3 months; its assessment was used in the present study, as well, to determine glycemic control. The mechanism improvement in glycemic control associated with aerobic exercise training may result in a decrease in oxidative stress. Aerobic exercise training improves insulin sensitivity [17, 18] and glycemic control [19]. The preferential loss of fat from the central regions during regular exercise is closely related to an improvement in insulin sensitivity [20], and weight loss in combination with decreased waist circumference may improve insulin sensitivity and glycemic control in our study.

The impact of exercise training on the lipid profile is variable. Lipid profile did not change baseline to twelve weeks, although several researchers have reported greather improvements with combining regular exercise and diet in lipid profile in type 2 diabetes [21, 22]. In the present study, aimed at regular, but moderate exercise, patients were instructed not to change their usual diet habits during the whole follow-up period, which may explain the disparity in the results.

We found decreases in blood pressure in aerobic exercise; and this phenomenon was also described by Gordon and colleagues who showed a significant reduction in systolic blood pressure after resistance training in patients with T2DM mellitus [23]. The beneficial effects on hypertension (blood pressure lowering, either systolic or diastolic) due to decreased activity of both the sympathetic nervous system and the renin-angiotensin system was also documented. Other mechanisms responsible for the antihypertensive effect of training include the decrease in peripheral arterial resistance (and elevated NO) caused by vasodilatation [24, 25].

MDA levels, as a marker of oxidative stress, showed highly significant relation with diastolic hypertension in our study. Lipid hydroperoxides decompose to form a variety of products including malondialdehyde. Malondialdehyde is used as an indicator of oxidative damage of cells and tissues. There is growing evidence that increased oxidative stress and associated oxidative damage are mediators of vascular injury in cardiovascular pathologies, including hypertension, and atherosclerosis [26]. Elevation of blood pressure in subjects with hypertension is accompanied by a marked increase in plasma and tissue lipid peroxidation product and an increase in MDA [26]. Increased blood pressure, shear stress, and blood flow have been shown to upregulate NOS expression [27]. This development has evoked considerable interest because of the possibilities that therapies targeted against reactive oxygen intermediates by decreasing generation of reactive oxygen species may be useful in minimizing vascular injury and hypertensive end organ damage. Regular exercise may protect against diseases associated with oxidative stress and hypertension.

In this study, lipid profile and soluble TNF-*α* remained essentially unaffected by twelve weeks of physical training. Previous studies examining the effects of exercise training on TNF-*α* levels have reported conflicting results. Some have reported increased and others have reported no changes in TNF-*α* levels after exercise training [8–11]. Interestingly, the current study did not observe any changes in TNF-*α* levels after exercise training. This suggests that the beneficial effects of exercise are not mediated via TNF-*α*.

In the present study, the levels of TNF-*α* did not change after the exercise training program, and we did not find a correlation between TNF-*α* and MDA or waist circumference. This discrepancy may be due to the smaller sample size of the present study. Therefore, larger and more diverse studies assessing changes in TNF-*α* and its relationship with MDA and waist circumference following exercise training are warranted. Our results suggest that T2DM mellitus subjects should be encouraged to increase their physical activity levels to prevent early development of chronic diseases related to diabetes mellitus.

## Figures and Tables

**Table 1:** Characteristics of aerobic exercise and control group at the baseline period (mean ± SD).

Variable	Aerobic exercise group (*n* = 31)	Control group (*n* = 33)	*P* value
Age, mean (yrs)	53.5 ± 6.5	54.0 ± 9.4	0.8
Gender	17 M/14 F	14 M/19 F	0.1
HbA1c (%)	6.8 ± 0.9	6.8 ± 0.8	0.8
BMI (kg/m^2^)	30.9 ± 4.6	30.6 ± 4.8	0.8
Waist circumference (cm)	101.9 ± 8.6	99.7 ± 10.7	0.3
Systolic arterial pressure (mmHg)	129.0 ± 12.9	124.1 ± 14	0.09
Diastolic arterial pressure (mmHg)	79.5 ± 7.1	78.0 ± 10.5	0.4
LDL-c (mg/dL)	106.4 ± 34.5	116.0 ± 35.5	0.1
HDL-c (mg/dL)	39.8 ± 9.1	41.3 ± 8.6	0.3
TG (mg/dL)	159.4 ± 90.2	149.9 ± 80.3	0.6
TC (mg/dL)	176.2 ± 42.0	190 ± 45.4	0.1
TNF-*α* (pg/mL)	14.2 ± 5.9	14.1 ± 5.1	0.9
MDA (nmol/mL)	6.56 ± 0.9	6.61 ± 1.0	0.8
Duration of diabetes (yrs)	6.2 ± 5.2	6.7 ± 4.5	0.6

BMI: body mass index, LDL-c: low density lipoprotein cholesterol, HDL-c: high density lipoprotein cholesterol, TG: triglyceride, TNF-*α*: tumor necrosis factor-alpha, MDA: malondialdehyde.

**Table 2:** Comparisons of clinical and laboratory characteristics baseline and after 12-week period in aerobic exercise and control group (mean ± SD).

Variable	Baseline aerobic exercise group (*n* = 31)	After 12-week period in aerobic exercise group (*n* = 31)	Baseline control group (*n* = 33)	After 12-week period in control group (*n* = 33)	*P* ^∧^ value	*P* ^*^ value
HbA1c (%)	6.8 ± 0.9	6.6 ± 1.0	6.8 ± 0.8	6.9 ± 1.1	0.1	0.4
BMI (kg/m^2^)	30.9 ± 4.6	30.4 ± 4.3	30.6 ± 4.8	30.5 ± 4.6	0.017	0.4
Waist circumference (cm)	101.9 ± 8.6	100.0 ± 8.2	99.7 ± 10.7	99.7 ± 10.5	0.012	0.7
Systolic arterial pressure (mmHg)	129.0 ± 12.9	122.5 ± 11.7	124.1 ± 14	124.3 ± 15.0	0.027	0.9
Diastolic arterial pressure (mmHg)	79.5 ± 7.1	75.8 ± 7.7	78.0 ± 10.5	77.0 ± 8.1	0.042	0.5
LDL-c (mg/dL)	106.4 ± 34.5	104.1 ± 32.1	116.0 ± 35.5	120.0 ± 31.5	0.7	0.6
HDL-c (mg/dL)	39.8 ± 9.1	40.9 ± 12.2	41.3 ± 8.6	43.0 ± 9.5	0.2	0.07
TG (mg/dL)	159.4 ± 90.2	144.0 ± 66.3	149.9 ± 80.3	164.0 ± 85.6	0.2	0.1
TC (mg/dL)	176.2 ± 42.0	172.0 ± 38.4	190 ± 45.4	193.0 ± 44.5	0.5	0.6
TNF-*α* (pg/mL)	14.2 ± 5.9	13.9 ± 5.2	14.1 ± 5.1	14.8 ± 5.7	0.7	0.2
MDA (nmol/mL)	6.56 ± 0.9	6.06 ± 1.0	6.61 ± 1.0	6.45 ± 0.8	0.046	0.2

BMI: body mass index, LDL-c: low density lipoprotein cholesterol, HDL-c: high density lipoprotein cholesterol, TG: triglyceride, TNF- *α*: tumor necrosis factor-alpha, MDA: malondialdehyde, *P* ^∧^ before versus after 12-week period in aerobic exercise group, *P* ^*^ before versus after 12-week period in control group.
